# [^18^F]Choline PET/CT and stereotactic body radiotherapy on treatment decision making of oligometastatic prostate cancer patients: preliminary results

**DOI:** 10.1186/s13014-016-0586-x

**Published:** 2016-01-22

**Authors:** Francesco Pasqualetti, Marco Panichi, Aldo Sainato, Fabrizio Matteucci, Luca Galli, Paola Cocuzza, Patrizia Ferrazza, Gabriele Coraggio, Giuseppe Pasqualetti, Lisa Derosa, Martina Sollini, Lorenzo Mannelli, Simona Ortori, Fabio Monzani, Sergio Ricci, Carlo Greco, Maria Grazia Fabrini, Paola Anna Erba

**Affiliations:** Radiation Oncology, Azienda Ospedaliero Universitaria Pisana, Via Roma 55, 56126 Pisa, Italy; Medical Oncology, Azienda Ospedaliero Universitaria Pisana, Via Roma 55, 56126 Pisa, Italy; Humanitas University, Via Manzoni 113, Rozzano, Milano, 20089 Italy; Geriatrics Unit, Azienda Ospedaliero Universitaria Pisana, Via Roma 55, 56126 Pisa, Italy; Radiology, Memorial Sloan-Kettering Cancer Center, 1275 York Avenue, New York, NY USA; Radiology, Azienda Ospedaliero Universitaria Pisana, Via Roma 55, 56126 Pisa, Italy; Radiation Oncology, Champalimaud Centre for the Unknown, Avenida Brasília, 1400-038 Lisboa, Portugal; Department of Translational Research and New Technologies in Medicine, Regional Center of Nuclear Medicine, University of Pisa, Via Savi 10, 56126 Pisa, Italy; Radiation Oncology, Pisa University Hospital, Via Roma 67, 56126 Pisa, Italy

**Keywords:** Choline PET/CT, Stereotactic body radiotherapy, Oligometastatic patients, Recurrent prostate cancer

## Abstract

**Background:**

A new entity of patients with recurrent prostate cancer limited to a small number of active metastatic lesions is having growing interest: the oligometastatic patients. Patients with oligometastatic disease could eventually be managed by treating all the active lesions with local therapy, i.e. either surgery or ablative stereotactic body radiotherapy. This study aims to assess the impact of [^18^F]Choline ([^18^F]FMCH) PET/CT and the use stereotactic body radiotherapy (SBRT) in patients (pts) with oligometastatic prostate cancer (PCa).

**Methods:**

Twenty-nine pts with oligometastatic PCa (≤3 synchronous active lesions detected with [^18^F]FMCHPET/CT) were treated with repeated salvage SBRT until disease progression (development of > three active synchronous metastases). Primary endpoint was systemic therapy-free survival measured from the baseline [^18^F]FMCHPET/CT.

**Results:**

A total of 45 lesions were treated with SBRT. After a median follow-up of 11.5 months (range 3–40 months), 20 pts were still in the study and did not receive any systemic therapy. Nine pts started systemic therapy, and the median time of the primary endpoint was 39.7 months (CI 12.20–62.14 months). No grade 3 or 4 toxicity was recorded.

**Conclusions:**

Repeated salvage [^18^F]FMCHPET/CT-guided SBRT is well tolerated and *could* defer the beginning of systemic therapy in selected patients with oligometastatic PCa.

## Background

Prostate cancer (PCa) is the most common malignant tumour in men in Western countries. Considering the progressive aging of the population, the clinical approach to this cancer needs to be redressed considering the presence of comorbidities and on-going medical treatments [1–3]. When the first-line curative strategies fail and the PSA value increases, the critical issue in asymptomatic PCa patients is the decision of whether to initiate systemic therapy, including androgen deprivation therapy (ADT) or chemotherapy [4, 5]. In recent years, improvements and availability of functional imaging modalities (e.g. radiolabeled choline PET/CT) have increased the detection of active metastatic lesions, even at low PSA level [6–8]. Therefore, by the increasing availability of radiolabeled choline PET/CT, a new entity of patients that present a limited number of active metastatic lesions is having growing interest: the oligometastatic patients [9–11]. Patients with oligometastatic disease could eventually be managed by treating all the active lesions with local therapy, i.e. either surgery or ablative stereotactic body radiotherapy (SBRT) [12–16]. Evidence on the safety and response rate of SBRT for the treatment of metastatic disease is growing, including the application in oligometastatic PCa [17, 18]. Despite the clinical and radiological response rate being described in more than 80 % of lesions treated with SBRT, target lesions to be treated and the most suitable population that would benefit from local treatment have yet to be established [19, 20]. However, the impact of radiolabeled choline PET/CT in selecting patients and validating the target lesions for subsequent SBRT has yet to be investigated.

The main and final outcome of this prospective study is the overall survival in patients with oligometastatic prostate cancer treated with *[*^*18*^F] Fluoro-Methyl Choline ([^18^F]FMCH) PET/CT-guided SBRT. Surrogate endpoints are the quality of life measured with EORTCT QLQ-C30, toxicity and the time to subsequent treatment after PSA recurrence detection [21]. The present manuscript reports the preliminary of this study.

## Methods

### Study design and population

**Fig. 1:**
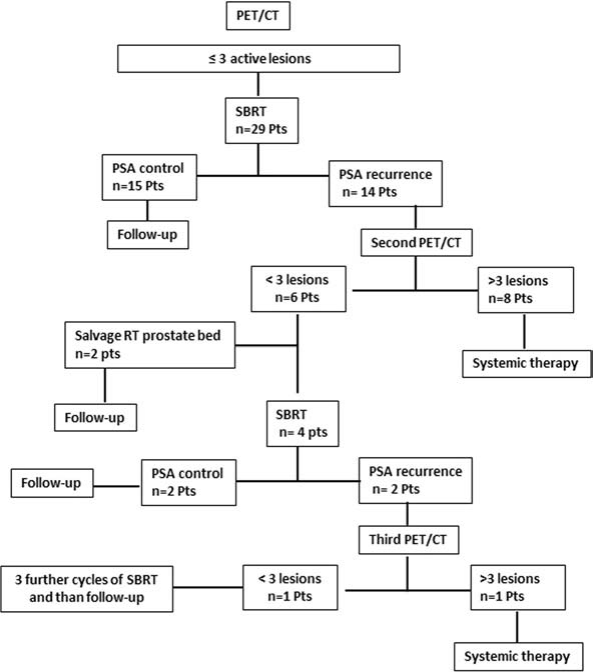
Study design

The study design is illustrated in Fig. 1.

Twenty-nine patients (median age of 71.2 years, range 50–79) with biochemical recurrence of prostate cancer and oligometastatic disease, defined as up to three synchronous (two or more lesions simultaneously revealed by the same PET/CT) active metastatic lesions as shown by [^18^F]FMCH uptake at PET/CT, were enrolled in the study and were treated with SBRT (Table 1). The study was approved by the local Institutional Review Board. Patient characteristics are reported in Table 1. Biochemical relapse was defined as two consecutive PSA values > 0.2 ng/ml in the case of patients treated with radical prostatectomy, or three consecutive increases in PSA following PSA nadir after primary External Beam Radiotherapy (EBRT), or three successive values demonstrating therapy failure in the case of castration-resistant PCa (CRPC) [4]. To exclude local relapse, all patients underwent endorectal Magnetic Resonance Imaging (*MRI)*. The median time from PCa diagnosis to study enrolment was 11.5 months (95 % CI 6.05–17.0 months). A multidisciplinary team discussed and approved all the cases included in this study.

**Table 1:** Main characteristics of the patients enrolled in this study

	Mean Value	SE
Age	69.86	1.24
PSA ng/ dL	3.43	1.05
Gleason Score	7.34	0.17
DT_mesi (months)	10.00	6.88
CTV (cm3)	2.97	0.57
	% Patients	
Castration Resistant	37.9	
Types of metastasis		
***Node lesions***	55.5	
*Obturator*	8.9	
*Iliac*	28.9	
*Parectal*	2.2	
*Lomboaortic*	13.3	
*Mediastinum*	2.2	
***Bone lesions***	44.5	
*Pelvis*	20.0	
*Vertebra*	8.9	
*Rib*	11.1	
*Sternum*	2.2	
*Clavicle*	2.2	

After SBRT, patients were followed-up by total PSA determination at 6-week and then every 3 months. Patients underwent clinical examination and comorbidities were recorded after each [^18^F]FMCHPET/CT scan, and every 3 months in the first 2 years of the study, and then every 6 months. In presence of biochemical recurrence after SBRT (two consecutive rises measured over 6 weeks), patients underwent a new [^18^F]FMCH-PET/CT scan. When [^18^F]FMCHPET/CT showed again the presence of oligometastatic disease, a further SBRT was performed, while when more than three active synchronous lesions were detected (plurimetastatic disease), patients were treated with systemic treatment in accordance to European Association of Urology (EAU) Guideline (hormonal treatment or chemotherapy), even in the presence of asymptomatic disease. Median patients’ follow-up was 11.53 months (95 % CI 6.05–17.02 months, range 3–40). No systemic treatment was delivered during study observation (patients who developed widespread diseases were switched to systemic therapy).

The primary endpoint of the study was the time between the baseline [^18^F]FMCH-PET/CT and the beginning of systemic therapy after the first SBRT, i.e. systemic therapy-free survival.

### Clinical examination

Medical history was recorded at enrolment, then the patients were evaluated before the first SBRT and at the end of the study in order to calculate the Cumulative Illness Rating Scale (CIRS) of comorbidity and their severity.

### Choline PET/CT

[^18^F]FMCH (IASOcholine® from IASON, Graz, Austria) was injected i.v.for the PET/CT scan (about 4 MBq/kg of body weight). As a routine protocol, imaging started 1 min after intravenous injection, with acquisition of dynamic PET images at one constant bed position of the pelvic region (covering the pelvic floor and urinary bladder) for 6 min (1 min per frame) to overcome the effect of urinary activity in the bladder. After this early phase, patients rested for approximately 1 h. The whole-body acquisition was performed thereafter in the three-dimensional mode, using 2 min per bed position from the base of the skull to the mid-thigh (six or seven bed positions). Images were reconstructed with a standard reconstruction ordered-subset expectation maximisation iterative algorithm (two iterative steps) and reformatted into transverse, coronal and sagittal views. Unenhanced CT was performed for localisation and attenuation correction (120 kV, 3.75 mm, pitch 0.984–0.5 s/rot spacing 3.27 mm - 47 slice, AutomA™ - SmartmA™ 15–100 mA Noise Index 25.03, 27-mm reconstructed section thickness). GE Discovery ST (GE Healthcare) was used for all scans.

PET/CT images were interpreted by two nuclear medicine specialists (PAE and LM) who were aware of the patient’s medical history and PSA values. Images were read sequentially using advanced PET/CT review software (Advantage for Windows, versions 4.2–7; GE Medical Systems), which allows simultaneous scrolling through the corresponding PET, CT and fusion images in transverse, coronal and sagittal planes. Semi-quantitative analysis of the abnormal radiotracer uptake was performed by using the maximum standardised uptake value (SUVmax).

### Diffusion weighted imaging (DWI), Dynamic contrast-enhanced (DCE-MRI)

All studies included imaging of the prostate with a dedicated endorectal coil (8-ch 1.5-T ID Medrad P/N M64ERA8-HD eCoil™ or 8-ch 3.0-T ID Medrad P/N M128ERA8-HD eCoil™) on a 3-T MR scanner, (GE Signa Excite HD 12.0 Twin-Speed 8-channel scanner, General Electric, Milwaukee, WI, USA) and a 1.5-T scanner (GE Signa Excite HD 12.0 Echospeed 8-channel scanner). Two radiologists aware of the clinical data, reviewed independently the images (SO and LM). The post-processing included mono- and bi-exponential ADC map calculation for prostate imaging. For the prostate imaging, DWI was fused with the T2 weighted images. Each MRI scan was classified as negative, positive or indeterminate for local relapse.

### Stereotactic body radiotherapy

SBRT treatment was performed using a Varian True Beam® platform and 6-MV photons with flattening filter-free beams. RapidArc®system was used for treatment planning. Axial CT images were obtained using a Light Speed RT 16-slice simulator (GE HealthCare). The CT image was set at 1.25-mm slices. Clinical target volume (CTV) was defined using [^18^F]FMCH-PET/CT scan imaging. Planning target volume consisted of both bone and nodal lesions of an isotropic 3-mm expansion of the CTV. Two different schedules of radiotherapy were delivered: 24 Gy in a single fraction or 27 Gy in three fractions, 2–3 sessions a week, were delivered (in the cases when it was not possible to meet the constraints for the single dose the dose of 27 Gy was delivered in 3 fractions). The delivered dose was prescribed at the periphery of the target. Before each fraction, a cone-beam CT was performed for patient’s set-up. Dexamethasone (4 mg) was administered 1 h after the SBRT session in the patients treated with 24 Gy in a single fraction. Task group 101 of the American Association of Physicists in Medicine constraints were used to avoid toxicity of the organs at risk [22]. Toxicity was recorded using the Common Terminology Criteria for Adverse Events (CTCAE) version 4.0 .

### Statistical analyses

All values are expressed as median and range, as is customary for non-parametric data. Survival probabilities without systemic therapy were estimated by the Kaplan-Meier method. Univariate Mantel-Cox analysis was used to examine the predictive value of covariates. All p-values were set at 0.05. Systemic therapy-free survival was considered the primary endpoint. Age, CTV volume, number of treated lesions, time to relapse, bone or nodal metastasis, the decline between the baseline PSA pre-SBRT and the PSA value 6 weeks after treatment (ΔPSA), CIRS severity and CIRS comorbidity were evaluated as possible covariates. Cases were censored at the beginning of systemic therapy or at the last follow-up visit if no systemic therapy was administered after SBRT. Statistical analysis was performed with SPSS v.15.0 (IBM Corp, Somers, NY, USA).

## Results

A total of 45 active lesions in 29 patients were detected by [^18^F]FMCH-PET/CT and treated with SBRT. [^18^F]FMCH-PET/CT identified 25 lesions involving lymph nodes and 20 bone lesions. No soft tissue, liver, adrenal or lung metastases were identified. Twenty-three lesions were treated with single dose radiotherapy and 22 with three fractions. Fifteen patients were treated for the presence of nodal disease, 12 for bone lesions and two patients presented both nodal and bone disease (Table 2). Fifteen out of 45 lesions were treated using a single arc while 30 lesions using 2 arcs. Nine patients started.

**Table 2:** Cox regression analysis of the main covariates of the time to systemic therapy form study entry for all analysed patients

	B	SE	Wald	df	Sig.	HR	HR 95,0 % CI	
							Lower	Upper
Age	0,11	0,06	3,56	1,00	0,06	1,12	1,00	1,25
Delta_PSA	0,00	0,00	4,50	1,00	0,03*	1,00	1,00	1,01
Castration Resistant	0,37	0,67	0,31	1,00	0,58	1,45	0,39	5,45
Gleason Score	−0,83	0,51	2,65	1,00	0,10	0,44	0,16	1,19
Metastasis (lynph node, bone, both)	−0,47	0,64	0,54	1,00	0,46	0,63	0,18	2,18
Doubling time (months)	0,00	0,02	0,01	1,00	0,91	1,00	0,96	1,05
Clinical target volume (CTV)	0.16	0.01	0.27	1,00	0.1	1,18	0,97	1,42
Cirs com	−1,12	0,70	2,57	1,00	0,11	0,33	0,08	1,28
Cirs sev	−3,20	2,67	1,44	1,00	0,23	0,04	0,00	7,66

*Delta PSA value resulted significant in the univariate analysis while it did not after adjusting for the age

**Fig. 2:**
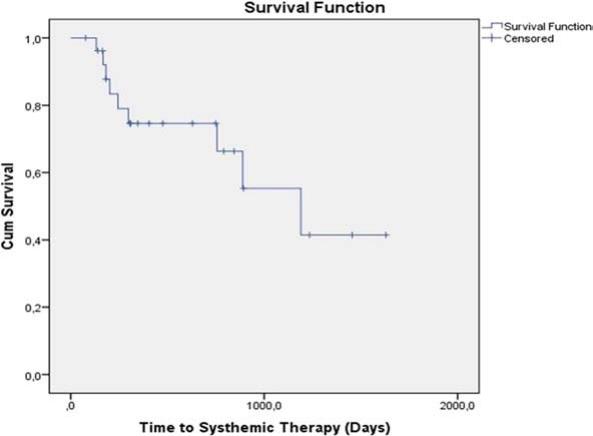
Cumulative survival from study entry to Follow up (event is the primary endpoint, start of systemic therapy)

Fifteen out of the 29 patients presented biochemical control after the first SBRT while 14 patients experienced PSA recurrence. Two of these patients developed a failure in the prostate bed after the first SBRT. They were both treated with salvage EBRT and because the achievement of total control PSA they were still considered oligometastatic and appropriate for this study. After a median follow-up of 11.5 months (range 3–40 months), 15/29 patients achieved a PSA control with a single course of SBRT, 2/29 patients with one course plus an EBRT for a prostate bed relapse, 2/29 patients with two courses of SBRT, one patients with three courses and one with five (Fig. 1). systemic therapy (in accordance with EAU Guideline) after a median time from the baseline [^18^F]FMCH-PET/CT of 39.7 months (primary endpoint, Fig. 2). All patients were alive at the time of data analysis. Grade 2, 3 or 4 toxicity was not recorded. Geriatric evaluation did not show any worsening during the course of the study.

**Fig. 3:**
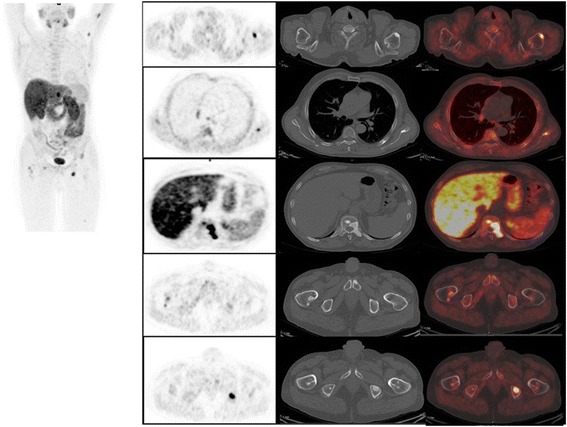
[^18^F]FMCH PET/CT and bone metastasis in a patient with wide spread disease

The median time of the primary endpoint was 39.7 months (95 % CI 17.2–62.1 months). Cox regression analysis of the selected covariates for the primary endpoint is reported in Table 2. CTV volume adjusted for age resulted associated with worse outcome, however this association did not reach the statistical significance (HR 1.197, 95 % CI 0.979–1.463, *p* = 0.08). Data relate to the quality of life require a longer follow-up to be interpreted.

### Pattern of failure

Patients who experienced PSA recurrence after the first SBRT presented the following pattern of relapse: two patients had disease progression on the prostate bed (treated with salvage EBRT). Four patients developed recurrence in lymph nodes and were still suitable for further SBRT. Eight patients presented recurrent disease classified as multi-metastatic disease and therefore underwent systemic therapy (seven patients ADT and one patient abiraterone acetate) (Fig. 3).

Recurrences have not been registered in the treated lesions. Interesting, six out of 10 patients with nodal recurrence and oligometastatic disease experienced relapse in a node in close proximity to the previously treated lymph nodes. Patients with bone metastasis, treated with SBRT, who experienced a further bone disease relapse, did not show any spread in the proximity of the treated lesion: they presented new distant metastasis in other bone segments. Finally, apart one case, we observed that patients with recurrent oligometastatic disease relapsed in the same tissue of the first diagnosed metastasis (bone-bone, node-node).

## Discussion

A critical issue of cancer treatment, and maybe a surrogate outcome to be investigated, might be the postponing of disease progression and therefore the beginning of subsequent therapy that can cause therapy-related symptoms [19, 20]. In recurrent PCa, PSA raise could be used to define disease progression without evidence of active lesions and it may be sufficient to declare the beginning of systemic therapy, either hormonal therapy or chemotherapy [23, 24]. In a prospective study, we decide to evaluate as a surrogate endpoint the delay of systemic therapy delivery in patients with hormone naïve and Castration resistance oligometastatic PCa.

The present paper reported the clinical outcomes of 29 patients with oligometastatic PCa treated with [^18^F]FMCHPET/CT-guided SBRT within a prospective single-institution clinical study. [^18^F]FMCH PET/CT was used for both patients and target lesions selection. We considered eligible for SBRT patients presenting high [^18^F]FMCH uptake in up to three lesions at PET/CT (oligometastatic disease). Even if the oligomestastatic state has been firstly described in 1995 by Hellamn S. et al. [9], few studies are available on the biochemical response related to the number of active lesions revealed by [^18^F]FMCH-PET/CT. On this basis, we choose to treat only patients with three active lesions in order to improve the chances to observe a clinical response. In our series, by treating all the active sites of disease with SBRT (delivering either 24 Gy in a single fraction or 27 Gy in three fractions), there was a significantly impact on patient treatment management. In particular, we obtained disease control, as shown by PSA levels, in 20 patients, thus avoiding the use of systemic therapy. In the remaining nine patients, we delayed the initiation of systemic therapy by 36 months.

Our results concerning the Systemic Therapy Free Survival are in line with evidence from the literature. In fact, in two studies with oligometaststic ADT-naïve PCa patients treated with SBRT, the use of functional imaging resulted in a deferral of systemic therapy by 38 and 25 months respectively, with a very high rate of disease control. In the present analysis, we measured a Systemic Therapy Free Survival of 39.7 months. This time was measured since the first choline PET/CT while in the other reported studies the ADT free survival was calculated since the first day of SBRT. However, in these studies PCa patients were selected by using [^*18*^F]FDG PET/CT and [^*18*^F]FMCHPET/CT was limited to few treated patients [12, 14]*.*

We considered oligometastatic and therefore suitable for local treatment, patients with less than 4 active lesions revealed by [^*18*^F]FMCHPET/CT. PSA value was not taken into account to prescribe a systemic therapy for patients enrolled without the evidence of more than three lesions. The patients enrolled into the studies conducted by Berkovic et al. and Decaestecker et al. started ADT if more than 3 active lesions were detected or in case PSA > 50 ng/mL (only for Berkovic et al. series) [12, 14]. In our series of PCa patients, CTV volume showed only a trend of recurrence prediction. None of the other covariates showed any predictive significance. Such findings underline the importance of a more accurate characterisation of the clinical and biochemical features of the “oligometastatic patients” that can benefit from local treatment on active sites of disease.

SBRT was able to achieve disease control independently from the site of disease relapse, with the response rates being virtually identical in cases of nodal disease and bone metastasis. In patients with nodal disease relapsed after SBRT and still presenting with an oligometastatic pattern of disease, the failure always occurred in nodes in close proximity to the previously treated lymph nodes, similar to that observed by Decaester et al. [14]. We suppose that such finding is of interest because it could be used to modify the SBRT approach in patients with nodal disease, providing a lower fractionated radiation dose on the lymph nodes of the involved chain. Micrometastatic disease is rarely detected by imaging modalities, thus leading to underestimation of disease burden in the closest lymph nodes [25]. According to the previous report [Decaester et al.], we found a consistent trend in the disease relapse pattern: patients with bone lesions tend to relapse in the bone tissues, while lymph node metastatic patients have proximity node recurrences. Understanding the clinical and biochemical features of this subgroup of patients could lead to the identification of the patients that are most likely to have disease progression throughout the lymphatic pathway and eventually eligible for prophylactic radiotherapy to the nodes closer to the site of [^18^F]FMCHPET/CT positive nodal recurrences.

## Conclusions

The preliminary results of this study indicated that the use of [^18^F]FMCHPET/CT-guided SBRT can significantly impact on treatment management of oligometastatic PCa patients, deferring the initiation of systemic therapy. A better identification of patients most likely to benefit from local therapy together with a deeper understanding of the reason of treatment failure are warranted before the introduction of this approach on a large scale.
